# Overexpression of thioredoxin m in tobacco chloroplasts inhibits the protein kinase STN7 and alters photosynthetic performance

**DOI:** 10.1093/jxb/ery415

**Published:** 2018-11-21

**Authors:** María Ancín, Alicia Fernández-San Millán, Luis Larraya, Fermín Morales, Jon Veramendi, Iker Aranjuelo, Inmaculada Farran

**Affiliations:** 1 Instituto de Agrobiotecnología (IdAB), Universidad Pública de Navarra-CSIC-Gobierno de Navarra, Campus Arrosadia, Pamplona, Spain; 2 Estación Experimental de Aula Dei (EEAD), CSIC, Departamento Nutrición Vegetal, Zaragoza, Spain

**Keywords:** LHCII phosphorylation, *Nicotiana tabacum*, photosynthesis, protein complex, thioredoxin, thylakoid membrane

## Abstract

The activity of the protein kinase STN7, involved in phosphorylation of the light-harvesting complex II (LHCII) proteins, has been reported as being co-operatively regulated by the redox state of the plastoquinone pool and the ferredoxin–thioredoxin (Trx) system. The present study aims to investigate the role of plastid Trxs in STN7 regulation and their impact on photosynthesis. For this purpose, tobacco plants overexpressing Trx f or m from the plastid genome were characterized, demonstrating that only Trx m overexpression was associated with a complete loss of LHCII phosphorylation that did not correlate with decreased STN7 levels. The absence of phosphorylation in Trx m-overexpressing plants impeded migration of LHCII from PSII to PSI, with the concomitant loss of PSI–LHCII complex formation. Consequently, the thylakoid ultrastructure was altered, showing reduced grana stacking. Moreover, the electron transport rate was negatively affected, showing an impact on energy-demanding processes such as the Rubisco maximum carboxylation capacity and ribulose 1,5-bisphosphate regeneration rate values, which caused a strong depletion in net photosynthetic rates. Finally, tobacco plants overexpressing a Trx m mutant lacking the reactive redox site showed equivalent physiological performance to the wild type, indicating that the overexpressed Trx m deactivates STN7 in a redox-dependent way.

## Teaser

Plants overexpressing chloroplast Trx m, but not f, inhibited STN7 activity in a redox-dependent way, which contributes to elucidation of the specificity of chloroplast Trxs for STN7 down-regulation.

## Introduction

The gathering of light energy and its later transformation into chemical energy is a central process for the proper functioning of photosynthetic machinery and plant performance. In photosynthetic organisms, light energy is captured by a set of light-harvesting complexes (LHCs) that constitute, together with their associated reaction centers and electron donors/acceptors, the PSI and PSII photosystems. Both photosystems are connected in series by a cytochrome *b*_6_*f* (Cyt*b*_6_*f*) complex. This connection allows the transport of electrons generated from the splitting of water in PSII towards the final electron acceptor of PSI, ferredoxin (Fd), all of which comprise the photosynthetic electron transport chain (ETC). Likewise, Fd provides electrons to key enzymes of the Calvin–Benson cycle for CO_2_ fixation via the Fd–thioredoxin (Trx) system, which carefully co-ordinates the light and carbon reactions of photosynthesis (Schürmann and Buchanan, 2008). As part of this system, chloroplast Trxs catalyze the reduction of disulfide bonds in target proteins, modulating their structure and function, and providing flexibility to plants for photosynthetic acclimation to changing environmental conditions (Nikkanen and Rintamäki, 2014; Geigenberger *et al.*, 2017). Recent reports highlight that while the two main chloroplast Trxs (m and f) are involved in Calvin–Benson cycle reduction *in vivo* (Okegawa and Motohashi, 2015; Naranjo *et al.*, 2016), Trx m seems to be more specifically engaged in the control of processes balancing photosynthetic reactions (Courteille *et al.*, 2013; Rey *et al.*, 2013; Wang *et al.*, 2013; Thormählen *et al.*, 2017; Da *et al.*, 2018).

Photosynthetic performance is highly regulated by environmental factors such as temperature, nutrients, and, in particular, light. Changes in light quality cause unequal distribution of excitation energy between the two photosystems. It is known that preferential excitation of PSII promotes the activation of a redox-sensitive kinase that allows the phosphorylation of LHCII. The phosphorylated LHCII (pLHCII) migrates from PSII towards PSI and shifts the excitation energy in favor of PSI (the so-called state 2). Conversely, under light conditions favoring PSI excitation, the kinase is deactivated and the pLHCII becomes dephosphorylated and relocated to PSII, thus increasing its cross-section and balancing the energy towards PSII (reversion to state 1). This rebalancing process, called state transitions, has been described as a fast post-translational acclimation mechanism operating in photosynthetic organisms under limiting light intensities (Allen, 1992, 2003; Lemeille and Rochaix, 2010; Tikkanen *et al.*, 2011). In vascular plants, LHCII comprises different homo- and heterotrimers of Lhcb1, Lhcb2, and Lhcb3 apoproteins (Galka *et al.*, 2012). According to their affinity for binding to the PSII core, LHCII trimers can be classified into at least three different types: S (strong), M (moderate), and L (loose) (Dekker and Boekema, 2005). Both S and M trimers play a minor role in state transitions, whereas the peripherally associated L trimer comprises the mobile fraction of the LHCII pool (Galka *et al.*, 2012; Wientjes *et al.*, 2013*a*; Crepin and Caffarri, 2015). Recent studies in Arabidopsis have demonstrated that S and M trimers still remain associated with PSII upon phosphorylation (Wientjes *et al.*, 2013*a*; Crepin and Caffarri, 2015), and that phosphorylated L trimers may also serve as an antenna for PSI in most natural light conditions (Wientjes *et al.*, 2013*b*), suggesting that association of pLHCII with PSI in higher plants may also represent a long-term response against changes in light intensity under most natural light conditions.

LHCII phosphorylation is triggered by the redox state of the plastoquinone (PQ) pool through the activation of a thylakoid-associated LHCII kinase (Bellafiore *et al.*, 2005; Bonardi *et al.*, 2005). Two orthologous LHCII kinases called Stt7 and STN7 have been identified in *Chlamydomonas* and Arabidopsis, respectively (Depège *et al.*, 2003; Bellafiore *et al.*, 2005). These are transmembrane proteins with catalytic domains exposed to the stroma that contain two conserved Cys residues, essential for their activity, located at the N-terminus on the lumenal side (Lemeille *et al.*, 2009; Wunder *et al.*, 2013*a*). The LHCII kinase requires an intact Cyt*b*_6_*f* complex to be active (Bennett *et al.*, 1988; Gal *et al.*, 1988, 1990) and is mainly regulated by the redox state of the PQ pool, with an interaction between plastoquinol and the quinol oxidation site of the Cyt*b*_6_*f* complex being critical for its activation (Vener *et al.*, 1997; Zito *et al.*, 1999). The interaction of Stt7/STN7 with the Cyt*b*_6_*f* complex occurs by means of the Rieske protein (PetC) (Lemeille *et al.*, 2009). Previous studies have shown that maximal Stt7/STN7 activity *in vivo* occurs at low light (LL) intensities, whereas it is drastically inhibited at higher irradiances (Schuster *et al.*, 1986; Rintamäki *et al.*, 1997). This inhibition has been reported to be mediated by the redox state of the chloroplast, most probably via the Fd–Trx system (Rintamäki *et al.*, 2000). The two well-conserved lumenal Cys residues have been reported to be the obvious target for stromal Trxs (Depège *et al.*, 2003), although they are located on opposite sides of the thylakoid membrane. If this were the case, a transthylakoid redox pathway would be required to make the kinase inactive (Dietzel *et al.*, 2008; Lemeille and Rochaix, 2010). Recent findings, however, have demonstrated that the disulfide bridge formed by the two conserved lumenal Cys residues is maintained during both activation and deactivation of the kinase (Shapiguzov *et al.*, 2016), indicating that mechanisms other than thiol reduction of these Cys residues should be involved in the regulation of the STN7 deactivation under high light (HL) conditions. Other authors have proposed the two conserved Cys residues located in the stroma as alternative substrates for Trxs (Rintamäki *et al.*, 2000; Puthiyaveetil, 2011). Rintamäki *et al.* (2000) postulated that the Trx target site of STN7 is hidden in the active kinase, whereas in HL it becomes exposed and thus is made available for Trx inhibition. However, these stromal Cys residues are conserved in land plants but not in unicellular green algae (Puthiyaveetil, 2011; Shapiguzov *et al.*, 2016) and, moreover, seem to not be required for STN7 activity and state transitions (Shapiguzov *et al.*, 2016). Therefore, the mechanistic basis for explaining the STN7 shut off through the Fd–Trx system is still the subject of debate.

The specificity of chloroplast Trxs in controlling the inactivation of STN7 under HL likewise remains largely unsolved. The first report on this, conducted with an *in vitro* phosphorylation assay of thylakoid membranes, involved both Trx f and m in the inhibition of LHCII phosphorylation, with Trx f being more efficient at low concentrations (Rintamäki *et al.*, 2000). Later, a direct physical interaction between STN7 and Trx f was demonstrated *in vitro* (Wunder *et al.*, 2013*b*). Recently, a plausible STN7 activation has been proposed in Arabidopsis *trxm1m2* mutants as a compensatory mechanism that allows increased photosynthesis during the LL periods of fluctuating light (Thormählen *et al.*, 2017). Here we have analyzed the specificity of chloroplast Trxs f or m in the redox regulation of STN7 and its impact on photosynthesis. This analysis was performed in wild-type (Wt) tobacco plants, as well as in lines overexpressing Trx f or m (o/exTrxf and o/exTrxm, respectively) from the plastid genome. Our findings demonstrate that overexpression of Trx m, but not Trx f, was associated with a complete loss of LHCII phosphorylation under LL conditions. In addition, the photosynthetic machinery was severely impaired in o/exTrxm plants. A putative role for Trx m in altering LHCII phosphorylation, and its consequences on modifying thylakoid architecture and photosynthetic performance in tobacco plants are discussed.

## Materials and methods

### Plant material and experimental conditions

Wt tobacco plants (*Nicotiana tabacum* cv. Petite Havana SR1) and plants overexpressing the mature *Trxf* or *Trxm* sequence from the chloroplast genome under the control of the P*rrnG10*L regulatory sequence (Sanz-Barrio *et al.*, 2013) were used in this study. Transformed and Wt plants were grown in a phytotron under the following conditions: 16 h light photoperiod, 80 µmol m^–2^ s^–1^ photosynthetic photon flux density (PPFD), and 28 ºC. Samples were taken from young fully expanded leaves of 7-week-old plants after 16 h light or 8 h dark, if not indicated otherwise. When necessary, plants were adapted to different light regimes: dark (D); LL at 80 µmol m^–2 –1^; HL at 800 µmol m^–2^ s^–1^ generated by a high-pressure sodium lamp (SON-T Agro 400; Philips, Amsterdam, The Netherlands); or far-red light (FR; which preferentially excites PSI) obtained by covering the sodium lamp with a Rosco-27 filter (Rosco Labs, Port Chester, NY, USA).

### Chl *a* fluorescence: fast transient and steady-state measurements

Fast transient Chl *a* fluorescence was measured using the portable FluorPen FP 100 (Photon Systems Instruments, Drasov, Czech Republic) in dark-adapted leaves (8 h) to allow the complete oxidation of reaction centers. Chl *a* fluorescence transients were induced by the exposure of plants to high irradiance (3000 µmol m^–2^ s^–1^), and fluorescence was recorded for 2 s. The data are shown as the relative fluorescence at time *t* (*Vt*), defined as (*F*_t_−*F*_o_)/(*F*_m_−*F*_o_).

Steady-state Chl *a* fluorescence measurements were carried out with the fluorometer of a Li-Cor 6400XT gas exchange portable photosynthesis system (Li-Cor, Lincoln, NE, USA) at a PPFD of 1200 µmol m^–2^ s^–1^. The quantum yield of PSII (Φ_PSII_) was calculated as (*F*_m_′–*F*_s_)/(*F*_m_′) (Genty *et al.*, 1989). Photochemical energy quenching (qP or qL) was calculated using either qP=(*F*_m_′–*F*_s_)/(*F*_m_′– *F*_o_′) or qL=(*F*_m_′–*F*_s_)/(*F*_m_′–*F*_o_′)×*F*_o_′/*F*_s_ (Kramer *et al.*, 2004). The fraction of closed (reduced) PSII reaction centers, also known as the excitation pressure (EP), was calculated as either 1–qP or 1–qL.

### Determination of gas exchange and photosynthetic electron transport rate

Fully expanded apical leaves were used to measure gas exchange with a Li-Cor 6400XT. The gas exchange response to [CO_2_] was measured by changing the [CO_2_] entering the leaf chamber with the following steps: 400, 300, 250, 200, 150, 100, 50, 400, 500, 600, 700, 800, 1000, 1200, and 1500 μmol mol^–1^, with 2–3 min between each step. The net rate of CO_2_ assimilation (*A*_N_), stomatal conductance (*g*_s_), transpiration rate (*E*), and substomatal CO_2_ concentration (C_i_) were estimated at a PPFD of 1200 µmol m^–2^ s^–1^ and 400 µmol mol^–1^ [CO_2_] using equations developed by von Caemmerer and Farquhar (1981). Estimations of the maximum carboxylation velocity of Rubisco (*V*c_max_), the maximum electron transport rate contributing to ribulose 1,5-bisphosphate (RuBP) regeneration (*J*_max_), and the triose phosphate utilization rate (TPU) were determined according to Sharkey *et al.* (2007).

Simultaneous measurements of Chl *a* fluorescence and CO_2_ exchange under non-photorespiratory conditions (2% O_2_ in volume) were performed with the Li-Cor 6400XT by varying the light intensity in a stepwise manner (from 2000 µmol m^–2^ s^–1^ to 0 µmol m^–2^ s^–1^). This procedure allows estimation of the rate of photosynthetic electron transport (ETR) according to Genty *et al.* (1989) as follows: ETR=4[(Φ_PSII_–*b*)/*a*]PPFD, with *a*, *b*, and PPFD being, respectively, the slope, the ordinate axis intercept of the relationship between Φ_PSII_ and Φ_CO2_, and the incident PPFD (Cornic and Briantais, 1991).

Finally, we calculated the distribution of energy between the two photosystems (*f*) from ETR=Φ_PSII_×PPFD×leaf absorptance×*f* (Kral and Edwards, 1992), assuming a maximum leaf absorptance of 0.875, based on the constancy of light absorption for the leaf Chl concentrations found in our experiments (Morales *et al.*, 1991).

### Chlorophyll content

Photosynthetic pigments were extracted from leaf disks collected from fully expanded leaves and crushed in 5 ml of 80% acetone. After centrifugation, the amount of Chl *a* and *b* was measured spectrophotometrically and calculated according to Lichtenthaler (1987).

### Detection of thylakoid phosphoproteins and the amount of STN7

Leaf samples (100 mg) were ground in liquid nitrogen, and thylakoid membranes were isolated according to Rintamäki *et al.* (1996). All of the extraction buffers contained 10 mM NaF (phosphatase inhibitor) to maintain the *in vivo* phosphorylation state. Protein concentration was measured using the RC protein assay (Bio-Rad, Hercules, CA, USA), according to the manufacturer’s instructions. Thylakoid extracts (15 µg of protein) were electrophoresed in a 15% polyacrylamide gel containing 6 M urea, and separated proteins were transferred to a polyvinylidene difluoride (PVDF) membrane for immunoblotting. Phosphoproteins were immunodetected using a rabbit polyclonal phosphothreonine antibody (Cell Signaling Technology, Danvers, MA, USA), at a dilution of 1:1000, and a peroxidase-conjugated goat anti-rabbit antibody (Sigma-Aldrich, St Louis, MO, USA) at a 1:10 000 dilution. Detection was performed using the ECL Prime western blotting detection reagent (GE Healthcare, Buckinghamshire, UK), according to the manufacturer’s instructions. To determine the amount of STN7 in these samples, blots were immunoprobed with a specific STN7 antibody (Agrisera AB, Vännäs, Sweden) at a dilution of 1:2000. Protein bands were detected using the ECL Select western blotting detection reagent (GE Healthcare).

### Structure and protein composition of thylakoid membranes

Thylakoid membranes, isolated as described before, were solubilized with 1.5% digitonin, a milder detergent that preserves weak interactions between protein complexes and provides information on protein complexes in the stroma lamellae as well as in grana margins and end membranes (Järvi *et al.*, 2011). Thylakoid proteins (80 µg) were analyzed by blue-native PAGE (BN-PAGE) using 4–16% NativePAGE Novex Bis-Tris gels (Invitrogen, ThermoFisher Scientific) according to the manufacturer’s instructions, which enables the resolution of protein complexes of high molecular weight (up to ≥1000 kDa). Thylakoid proteins were then transferred to a PVDF membrane, immunoprobed against Lhcb1 and Lhcb2 antibodies (1:1000 dilution; Agrisera AB), and detected by ECL Prime.

For TEM analysis, leaf samples from Wt and o/exTrxm plants were fixed and processed as previously described (Sanz-Barrio *et al.*, 2011).

### Pull-down of His-tagged proteins

Chloroplasts from tobacco plants were isolated (Fernández-San Millán *et al.*, 2018) and protein–protein interactions were stabilized *in situ* using the cell-permeable DSP [dithiobis(succinimidyl propionate)] cross-linker (ThermoFisher Scientific). Cross-linked chloroplasts were lysed [50 mM BisTris, 6 N HCl, 50 mM NaCl, 10% (w/v) glycerol, 1% digitonin, and protease inhibitor cocktail], and His-tagged Trx target complexes were pulled down using Ni-NTA agarose (Qiagen, Hilden, Germany) as follows: chloroplast lysates were incubated with 200 µl of Ni-NTA resin for 3–4 h at 4 °C. After centrifugation at 600 *g* for 1 min, the supernatant was discarded and the pellet was washed five times with washing buffer (lysis buffer without digitonin). The pulled-down proteins were eluted with 4× Laemmli buffer (0.5 M Tris–HCl pH 6.5, 4% SDS, 20% glycerol, and 10% β-mercaptoethanol). Input extracts and pulled-down proteins were analyzed by SDS–PAGE and immunoblotted with specific antibodies: anti-STN7 (Agrisera AB), anti-Trx m and anti-Trx f (Sanz-Barrio *et al.*, 2011), anti-2-Cys peroxiredoxin (Prx) (Pérez-Ruiz *et al.*, 2006), or anti-Lhcb1.

## Results

### Overexpression of Trx m, but not f, impedes LHCII phosphorylation by down-regulating STN7 activity

**Fig. 1.**
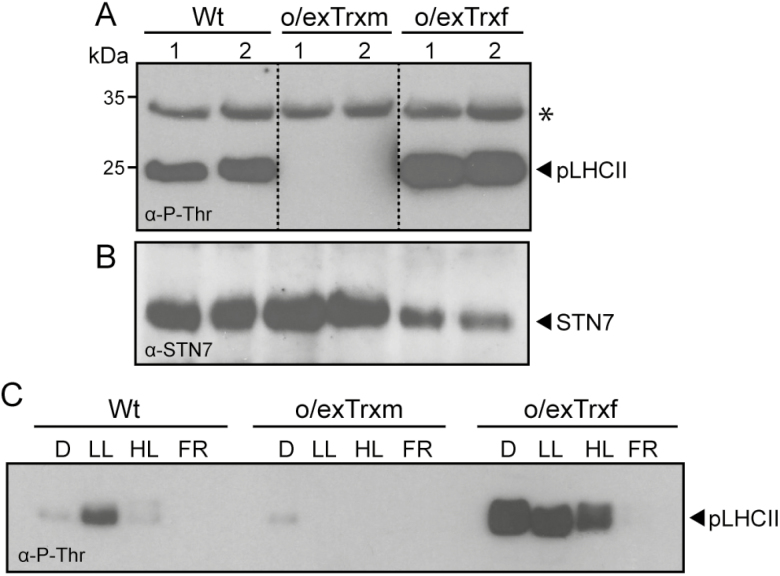
Effect of Trx m or f overexpression in tobacco chloroplasts on thylakoid protein phosphorylation and STN7 accumulation. (A) Thylakoid protein phosphorylation in Wt, o/exTrxm, and o/exTrxf plants sampled during the light (16 h at 80 μmol m^−2^ s^−1^) period. Thylakoid proteins (15 µg) were separated by SDS–PAGE (15%+6 M urea), transferred to a PVDF membrane, and immunoblotted with a phosphothreonine antibody. Phosphorylated LHCII (pLHCII) proteins are indicated. The asterisk represents phosphorylated PSII core proteins (D1 or D2). Molecular weights (kDa) are indicated on the left. (B) STN7 protein accumulation in the samples described in (A). The PVDF membrane was probed with an antibody raised against STN7. (C) LHCII phosphorylation pattern under different light regimes. Wt, o/exTrxm, and o/exTrxf plants were placed in darkness (D) for 8 h and then transferred for 2 h to low light (LL; 80 μmol m^−2^ s^−1^), followed by exposure to high light (HL; 800 μmol m^−2^ s^−1^) or far red light (FR) for 1 h. At the end of light treatments, the LHCII phosphorylation of isolated thylakoids was analyzed by western blot.

The phosphorylation status of isolated thylakoids from Wt, o/exTrxf, and o/exTrxm plants grown under LL conditions (80 µmol m^–2^ s^–1^) was monitored by western blot (Fig. 1A). LHCII phosphorylation, which is predominantly mediated by STN7, was detected in Wt and o/exTrxf plants, whereas it was completely lacking in o/exTrxm plants. In contrast, levels of phosphorylated PSII core proteins (regulated by the protein kinase STN8; Vainonen *et al.*, 2005) barely differed among o/exTrxf, o/exTrxm, and Wt plants. These results indicate that the overexpression of Trx m in tobacco plants specifically inhibits the phosphorylation status of STN7-specific substrates. Curiously, STN7 was found at even higher levels in o/exTrxm plants than in Wt and o/exTrxf plants (Fig. 1B), which indicates that the lack of phosphorylation in o/exTrxm plants did not correlate with a decrease in the amount of STN7, but instead may be indicative of a down-regulation of its kinase activity.

To compare the LHCII phosphorylation pattern under different light regimes, Wt, o/exTrxf, and o/exTrxm plants were left in darkness for 8 h, and then placed for 2 h in LL, which normally induces state 2. Plants were then exposed to 1 h of either HL or FR conditions to induce state 1. As expected, LHCII phosphorylation in Wt plants was inhibited in darkness and became activated under LL conditions, whereas treatments with HL or FR again triggered complete dephosphorylation of LHCII (Fig. 1C). Interestingly, o/exTrxm plants seemed to be arrested in state 1, with LHCII constitutively dephosphorylated. In contrast, o/exTrxf plants retained the LHCII phosphorylation under dark and HL conditions as if they were trapped in state 2 (Fig. 1C). Subsequently, only a prolonged FR exposure (1 h) allowed the transition from state 2 to 1.

### The redox status of the PQ pool is altered in Trx-overexpressing tobacco plants

**Fig. 2.**
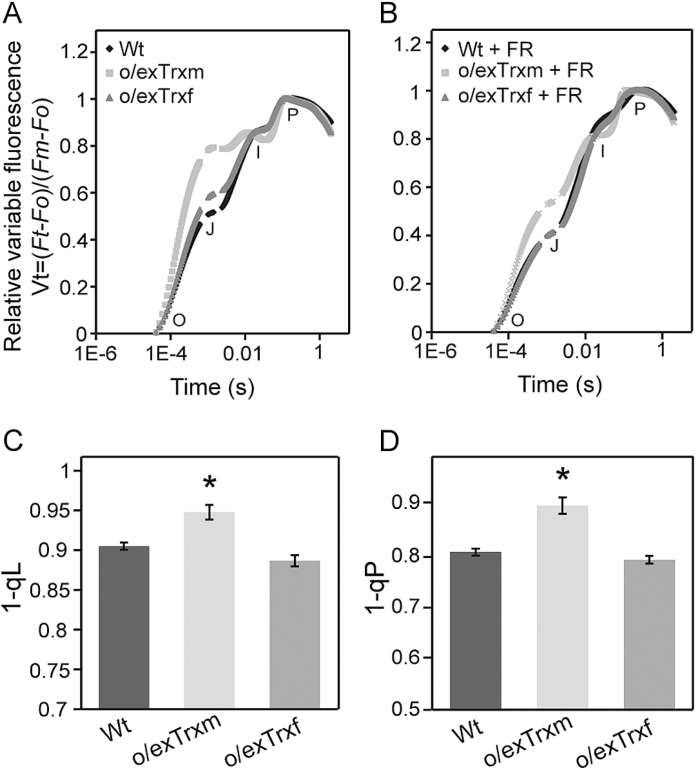
Effect of Trx overexpression on Chl *a* fluorescence transients in o/exTrxm, o/exTrxf, and Wt tobacco leaves. O, J, I, and P points represent increasing Chl fluorescence values during exposure to a short saturating light pulse. (A) Normalized Chl *a* fluorescence transients of overnight dark-adapted leaves. (B) Normalized Chl *a* fluorescence transients of overnight dark-adapted leaves subjected to a 1 min far-red (FR) pulse followed by 30 s of dark adaptation. (C) Redox status of the PQ pool expressed as 1–qL. (D) Redox status of the PQ pool expressed as 1–qP. Data shown are the mean ±SE (*n*=6 plants for each line). Statistical significance compared with Wt plants is indicated by asterisks (*P*<0.05, Student’s *t*-test).

It is well known that STN7 activation is controlled by reductions in the PQ pool. The fast transient of Chl *a* fluorescence (OJIP; Strasserf *et al.*, 1995) has been used to estimate the redox state of the intermediate electron carriers in the transgenic lines. As shown in Fig. 2A, the shape of the OJIP transient rise was altered in dark-adapted leaves of o/exTrxf and o/exTrxm plants, which exhibited an increase in the J value of ~15% and 60% compared with Wt plants, respectively. Hence, in both transgenic lines, the area above the J–I curve was smaller than in the Wt (Fig. 2A). This area is assumed to be a measure of the number of oxidized PQ molecules available at the beginning of the fluorescence measurement (Tóth *et al.*, 2005), suggesting that the fraction of reduced PQ in the dark is increased in both transgenic plants relative to the Wt. To corroborate this fact, Wt, o/exTrxf, and o/exTrxm leaves were subjected to FR as a way of removing the electron accumulation in the PSII acceptor side (Belkhodja *et al.*, 1998). Leaves were therefore subjected to a 1 min FR pulse followed by 30 s of dark adaptation to avoid any possible actinic effect induced by the FR pulse. After this treatment, the OJIP transient of dark-adapted Trx-overexpressing leaves almost recovered the Wt shape (Fig. 2B), which confirms the over-reduction of the PQ pool in both transgenic lines. To investigate the redox state of the PQ pool in light, the fluorescence parameters 1–qL and 1–qP were used. As shown in Fig. 2C and D, both parameters were significantly increased in o/exTrxm plants, which indicates that a more reduced PQ pool also occurs at saturating light conditions and suggests that the PQ pool would be permanently over-reduced in this genotype. Therefore, the decreased STN7 activity in o/exTrxm plants under LL does not appear to correlate with an oxidation of the PQ pool.

### Overexpression of Trx m alters the structure and protein composition of thylakoid membranes

**Fig. 3.**
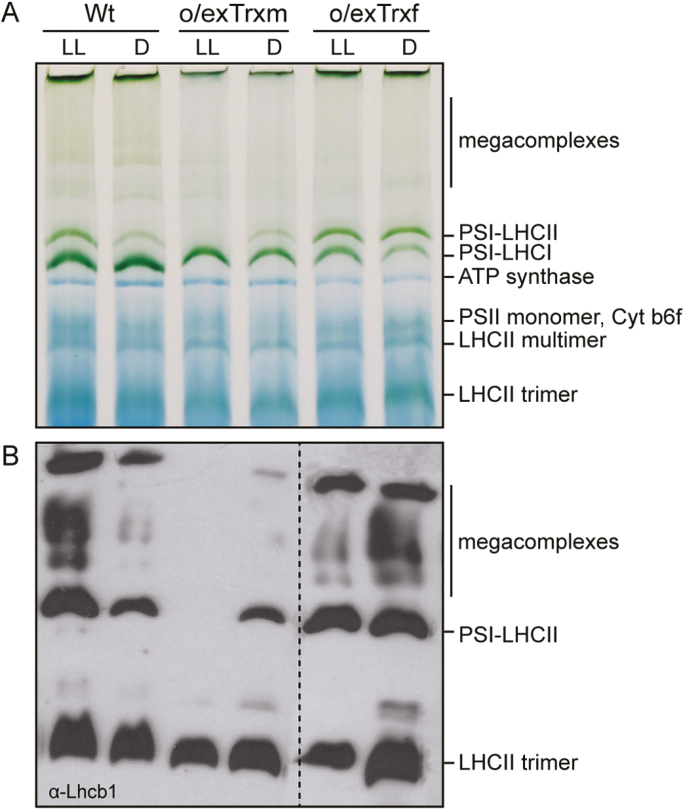
Thylakoid protein complex organization and composition. (A) Thylakoid protein complexes (80 µg) from Wt, o/exTrxm, and o/exTrxf plants were solubilized with 1.5% digitonin and separated by BN-PAGE. Identification of protein complexes was performed in accordance with Järvi *et al.* (2011) and Wunder *et al.* (2013*b*). (B) Lhcb1 protein content in thylakoid complexes. Thylakoid complexes reported above were transferred to a PVDF membrane and immunoblotted against Lhcb1 antibody.

To assess the effect of inhibited LHCII phosphorylation on formation of the PSI–LHCII supercomplex during state transitions, BN-PAGE analyses were performed on digitonin-solubilized thylakoid membranes from LL- and dark-adapted Wt, o/exTrxm, and o/exTrxf plants (Fig. 3). The state transition-specific PSI–LHCII supercomplex appeared in Wt thylakoids that were exposed to LL (state 2), whereas it was markedly reduced in Wt thylakoids kept in darkness (state 1) (Fig. 3A). The BN-PAGE analysis revealed no significant differences between Wt and o/exTrxf under LL conditions, while the PSI–LHCII supercomplex failed to form in o/exTrxm thylakoids (Fig. 3A). This indicates that the absence of phosphorylation in the o/exTrxm line (Fig. 1C) impedes migration of LHCII from PSII to PSI, with the concomitant loss of PSI–LHCII supercomplex formation. Conversely, a large portion of the PSI–LHCII supercomplex persisted in thylakoid membranes from dark-adapted o/exTrxf leaves (Fig. 3A), which is consistent with the high LHCII phosphorylation level shown by this line in darkness (Fig. 1C).

The mobile LHCII trimers performing state transitions are mainly composed of Lhcb1 and Lhcb2 proteins (Galka *et al.*, 2012). We therefore analyzed the level of these two apoproteins in thylakoid extracts. BN-PAGE combined with immunoblotting showed that in the Wt, Lhcb1 and 2 mainly migrated as LHCII trimers but also as PSI–LHCII complexes and larger megacomplexes under LL, whereas the abundance of these complexes decreased in the dark (Fig. 3B; Supplementary Fig. S1 at *JXB* online). The larger digitonin-solubilized megacomplexes mainly represented PSI–LHCII megacomplexes and PSI–PSII complexes migrating together (Järvi *et al.*, 2011). Trx f-overexpressing plants exposed to LL displayed a similar Lhcb1 and 2 distribution to the Wt, while the number of state transition-specific complexes considerably increased in thylakoids of o/exTrxf plants kept in the dark. In contrast, Lhcb1 and 2 proteins were not detected at the gel position corresponding to the PSI–LHCII complex or at the location of the larger megacomplexes in o/exTrxm plants grown under LL (Fig. 3B; Supplementary Fig. S1). Taken together, our results support the idea that phosphorylation of LHCII proteins strongly influences the formation of such megacomplexes.

**Fig. 4.**
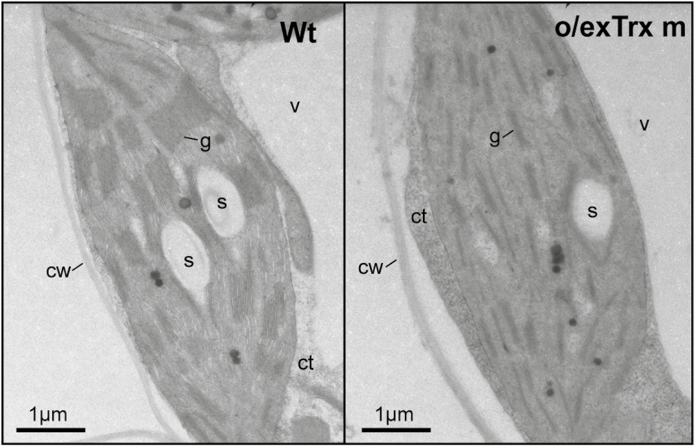
Thylakoid ultrastructure of o/exTrxm plants. TEM was performed to examine leaf mesophyll cells from tobacco Wt and Trx m-overexpressing plants. Representative cross-sections of chloroplasts are shown. v, vacuole; ct, cytoplasm; cw, cell wall; g, grana; s, starch.

The overexpression of Trx m in tobacco plants altered not only the phosphorylation pattern of Lhcb 1 and 2 apoproteins, but also their abundance in chloroplasts. An Lhcb1/2 reduction of ~50% was observed in o/exTrxm compared with Wt and o/exTrxf plants (Supplementary Fig. S2). To examine whether changes in the LHCII abundance could influence thylakoid ultrastructure, chloroplasts of Wt and o/exTrxm plants were analyzed by TEM. As shown in Fig. 4, thylakoids from Wt plants formed a continuous network of stromal lamellae with stacked grana (left panel), whereas thylakoids from o/exTrxm plants were partially unstacked, with stacks exhibiting fewer membrane layers (reduction in grana height) than in the Wt (right panel).

### Overexpression of Trx m negatively affects photosynthesis

Gas exchange analyses conducted in leaves of Wt, o/exTrxm, and o/exTrxf plants showed no differences between the Wt and o/exTrxf (Table 1). On the other hand, while no significant differences were detected in stomatal opening (*g*_s_) and leaf transpiration (*E*) between the three genotypes, the net photosynthesis (A_N_) strongly decreased in o/exTrxm plants. The higher substomatal CO_2_ concentration (C_i_) of o/exTrxm may be linked with their lower CO_2_ fixation rates. *A*–C_i_ curve analyses revealed that depleted photosynthetic rates detected in o/exTrm were associated with the lower values detected for the maximum carboxylation velocity of Rubisco, the maximum electron transport rate contributing to RuBP regeneration, and the triose phosphate utilization rate (*V*c_max_, *J*_max_, and TPU, respectively).

**Table 1.** Net photosynthesis (*A*_N_, µmol CO_2_ m^–2^ s^-1^), substomatal CO_2_ concentration (C_i_, µmol CO_2_ mol^–1^ air), stomatal conductance (*g*_s_, mol H_2_O m^–2^ s^–1^), transpiration (*E*, mmol H_2_O m^–2^ s^–1^), maximum carboxylation velocity of Rubisco (*V*c_max_, µmol m^–2^ s^–1^), maximum electron transport rate contributing to RuBP regeneration (*J*_max_, µmol m^–2^ s^–1^), and triose phosphate utilization rates (TPU, µmol m^–2^ s^–1^) in 7-week-old wild-type, and Trx m- and f-overexpressing plants (Wt, o/exTrxm, and o/exTrxf, respectively)

	*A*_N_	C_i_	*g*_s_	*E*	Vc_max_	*J*_max_	TPU
Wt	11.69 ± 0.47 a	292.02 ± 5.67 b	0.208 ± 0.021 a	2.83 ± 0.45 a	56.56 ± 3.86 a	66.64 ± 1.85 a	3.69 ± 0.14 a
o/exTrxm	6.99 ± 0.34 b	326.87 ± 7.55 a	0.210 ± 0.023 a	3.27 ± 0.33 a	32.28 ± 1.22 b	38.42 ± 1.38 b	2.09 ± 0.13 b
o/exTrxf	10.99 ± 0.36 a	294.59 ± 9.07 b	0.204 ± 0.017 a	2.93 ± 0.35 a	49.47 ± 2.79 a	61.97 ± 1.65 a	3.25 ± 0.12 a

Values are means ±SE (*n*=6–9). Different letters denote significantly different values (ANOVA, *P* <0.05).

Effects of Trx m overexpression on photochemistry were further confirmed by simultaneous measurements of the light response curves of photosynthesis and fluorescence at low O_2_, which allows quantification of photosynthetic ETR in the absence of photorespiration. The data revealed a lower photosynthetic ETR in o/exTrxm than in the Wt plants (Table 2), in line with the above-mentioned decreased *J*_max_ values. In addition, this approach allows estimation of the light distribution between PSII and PSI. These results indicated a lower distribution of light to PSII in o/exTrxm than in the Wt plants (Table 2), suggesting changes in the stoichiometry of PSII with respect to PSI in o/exTrxm plants. The Chl *a* and *b* concentrations were also reduced in o/exTrxm compared with Wt and o/exTrxf plants (Table 2). Moreover, significant differences in the Chl *a*/*b* ratio were found between lines, the ratio being higher in o/exTrxm plants as a consequence of a more prominent decrease in Chl *b* than in the Chl *a* concentration (Table 2). This result can be explained by decreases in antenna size and/or alterations in the stoichiometry of thylakoid pigment–protein complexes of the photosystems. The latter would be in agreement with the described alteration of light distribution between photosystems.

**Table 2.** Chl *a* and *b* concentration (µg cm^–2^), Chl *a/b* ratio, photosynthetic electron transport rate (ETR, µmol e^–^ m^–2^ s^–1^), and energy distribution between PSII and PSI (*f*) in 7-week-old wild-type, and Trx m- and f-overexpressing plants (Wt, o/exTrxm, and o/exTrxf, respectively)

	Chl *a*	Chl *b*	Chl *a/b*	ETR	*f*
**Wt**	27.77 ± 1.38 a	8.73 ± 0.30 a	3.18 ± 0.08 b	62.50 ± 1.45 a	0.54 ± 0.00 a
**o/exTrxm**	13.67 ± 0.48 b	3.80 ± 0.08 b	3.60 ± 0.12 a	30.67 ± 2.05 b	0.46 ± 0.00 b
**o/exTrxf**	26.97 ± 1.37 a	8.51 ± 0.67 a	3.17 ± 0.12 b	ND	ND

Values are means ±SE (*n*=6–9). Different letters denote significantly different values (ANOVA, *P* <0.05).

ND, not determined.

### Tobacco plants overexpressing the Trx m redox mutant variant recovered the Wt phenotype

**Fig. 5.**
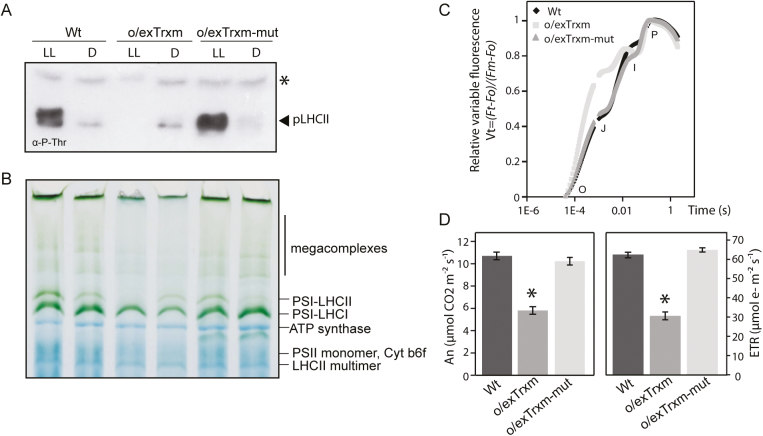
Recovery phenotype in tobacco plants overexpressing the redox mutant variant of Trx m. (A) LHCII protein phosphorylation in Wt, o/exTrxm, and o/exTrxm-mut plants sampled under low light (LL, 80 μmol m^−2^ s^−1^) or dark (D) conditions. Thylakoid proteins (15 µg of protein) were separated by SDS–PAGE (15%+6 M urea), transferred to a PVDF membrane, and immunoblotted with a phosphothreonine-specific antibody. The asterisk represents phosphorylated PSII core proteins (D1 or D2). (B) Pigment–protein complexes from thylakoids (80 µg) were separated by BN-PAGE. The identity of protein complexes is shown. (C) Normalized OJIP transients of overnight dark-adapted leaves. (D) Net rate of CO_2_ assimilation (*A*_N_) and the photosynthetic electron transport rate (ETR).

To ascertain whether the STN7 inactivation observed in o/exTrxm plants was triggered by the reductase activity of the overexpressed Trx m, the two Cys residues within its catalytic domain were replaced by serine (C37/40S) to generate the redox mutant. Transplastomic tobacco plants overexpressing this mutant (o/exTrxm-mut) were generated by chloroplast transformation as previously described (Sanz-Barrio *et al.*, 2013), and integration of the transgene into plastid DNA and homoplasmy was confirmed (Supplementary Fig. S3A, B). O/exTrxm-mut plants almost recovered the Wt phenotype, with the expression level of the mutated variant being similar to that of Trx m in o/exTrxm plants (Supplementary Fig. S3C, D). Regarding the LHCII phosphorylation status, pLHCII was detected in LL-adapted o/exTrxm-mut plants at similar levels to those in the Wt (Fig. 5A), which indicates that STN7 conserves its phosphorylation capacity in this line. Accordingly, BN-PAGE analyses of thylakoid protein complexes clearly revealed that o/exTrxm-mut plants also recovered the state transition-specific PSI–LHCII complex under LL conditions (Fig. 5B). When the fast Chl *a* fluorescence transient was analyzed in leaves of o/exTrxm-mut plants, the OJIP shape shifted towards that of the Wt plants (Fig. 5C). In agreement with these findings, the *A*_N_ and ETR in o/exTrxm-mut plants reached values similar to those of Wt plants (Fig. 5D). Taken together, our results provide evidence for the recovery of the Wt phenotype when the Trx m redox mutant was overexpressed in tobacco chloroplasts, suggesting that, in o/exTrxm plants, the overexpressed Trx m inhibits STN7 activity and impairs the photosynthetic performance of tobacco plants in a redox-dependent way.

### Putative protein–protein interaction between Trx m and STN7/PetC proteins

**Fig. 6.**
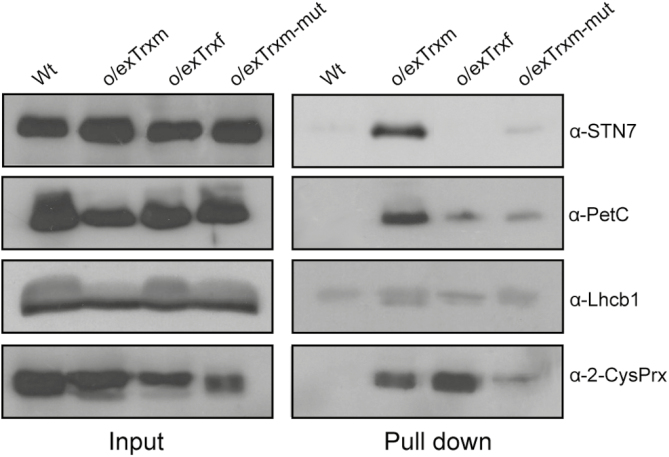
*In vivo* pull-down assay showing interaction between Trx m and STN7/PetC. Protein complexes from Wt and His-tagged o/exTrxm, o/exTrxf, and o/exTrxm-mut cross-linked chloroplasts were pulled-down with Ni-NTA resin. After washing the beads, bound proteins were eluted by boiling and analyzed, together with input fractions, by western blot using anti-STN7, anti-PetC, anti-2-Cys Prx, and anti-Lhcb1 antibodies.

To investigate a putative protein–protein interaction between Trx m and STN7 or proteins involved in the kinase activation such as PetC, an *in vivo* pull-down assay using chloroplasts from His-tagged Trx m-overexpressing tobacco plants was performed. Chloroplasts from Wt, o/exTrxf, and o/exTrxm-mut were used as controls. The pull-down assay allowed recovery of substantial amounts of overexpressed Trxs, which correlated with the amount present in the respective input extracts (Supplementary Fig. S4). Input extracts and pulled-down proteins were analyzed by SDS–PAGE and then immunoblotted with anti-STN7 and anti-PetC (Fig. 6). The results indicated that STN7 and PetC were barely detected in the pulled-down fraction from Wt, o/exTrxf, and o/exTrxm-mut plant extracts, whereas they were enriched in the fraction from o/exTrxm extracts (Fig. 6). The input and pulled-down fractions from each line were also immunoblotted with anti-2-Cys Prx, a well-known Trx target protein (König *et al.*, 2002), and anti-Lhcb1 (a non-target protein) as positive and negative controls, respectively. While no presence of Lhcb1 was found in any pulled-down fraction, 2-Cys Prx co-precipitated with both the Trx m and f proteins (Fig. 6). Altogether, these results demonstrate a putative protein–protein interaction, either directly or through associated partners, between Trx m and the STN7/PetC proteins.

## Discussion

### Phosphorylation pattern of LHCII proteins in Trx-overexpressing tobacco plants

Our study highlighted that, under typical inhibitory light conditions (such as HL and darkness; Rintamäki *et al.*, 1997), the overexpression of Trx f in tobacco chloroplasts is associated with an induction of LHCII phosphorylation (Fig. 1). In fact, pLHCII levels in the o/exTrxf line were comparable with (under HL) or even higher (in the dark) than those induced by LL. Similar results were reported in transgenic Arabidopsis lines overexpressing STN7 (Wunder *et al.*, 2013*a*). However, in our case, differences in the LHCII phosphorylation pattern between o/exTrxf and Wt plants could not be explained by an increase in the STN7 amount since it was even lower than in the Wt (Fig. 1B). Indeed, according to Hou *et al.* (2002), regulation of LHCII phosphorylation might be due not only to light conditions but also to the metabolic status of the chloroplast. In particular, increased LHCII phosphorylation in darkness has been observed in Arabidopsis by feeding leaves with glucose (Tikkanen *et al.*, 2010). Previous studies have shown that Trx f overexpression in tobacco chloroplasts increases the soluble sugar content in leaves, and particularly that of glucose (Sanz-Barrio *et al.*, 2013). Therefore, we presume that the STN7 activation observed in o/exTrxf tobacco plants could be due to the metabolic status of the chloroplast. More specifically, the sugar accumulation would favor production of NADPH from the oxidative pentose phosphate pathway, leading to a non-photochemical reduction of the PQ pool (Fig. 2A, B) via NAD(P)H dehydrogenase (Corneille *et al.*, 1998) and the subsequent STN7 activation. In agreement with this, an increase in the NADPH/NADP ratio was recorded in this transgenic line (Supplementary Fig. S5). The over-reduced PQ pool in o/exTrxf plants is only apparent in OJIP measurement but not in 1–qL and 1–qP (Fig. 2C, D), probably because the pressure of electron flow at the saturating light conditions employed for these measurements may overcome the PQ pool over-reduction in this genotype.

In o/exTrxm plants, however, STN7 seems to be insensitive to the reduction state of the PQ pool (Figs 1, 2), and the pattern of LHCII phosphorylation resembles that of *stn7* mutants (Wunder *et al.*, 2013*a*). Nevertheless, the lack of LHCII phosphorylation in o/exTrxm leaves could not be explained by a down-regulation of the amount of STN7, but rather STN7 tends to accumulate at even higher levels than in the Wt (Fig. 1B). It is known that STN7 abundance is regulated at both the transcriptional and post-translational level in a light- and redox-dependent manner (Wunder *et al.*, 2013*a*). Accordingly, the STN7 accumulation in o/exTrxm plants clearly correlated with a more reduced PQ pool (Figs 1, 2). The PQ pool over-reduction in o/exTrxm plants may be linked to a decrease in the PSI activity, mainly caused by the lack of state 2 transition (i.e. smaller PSI antenna size) and decreased Fd amount at the PSI acceptor side (Supplementary Fig. S6). An over-reduction of transthylakoid redox carriers such as CcdA and HCF164, caused by Trx m overexpression (discussed below), could likewise cause an unbalanced PQ pool. The STN7 accumulation in o/exTrxm plants was, however, not accompanied by an increase in the STN7 kinase activity, as reported for several photosynthetic mutant lines with constitutively over-reduced PQ pools (Wunder *et al.*, 2013*a*), but instead resulted in a complete STN7 deactivation (Fig. 1). The increase in STN7 levels in o/exTrxm plants could therefore be a compensatory response to the absence of state transitions. Similar results were reported in Arabidopsis mutants lacking Lhcb1, where STN7 also tends to accumulate in the absence of state transitions (Pietrzykowska *et al.*, 2014).

In summary, we demonstrated *in vivo* that the overexpression of Trx m, but not its counterpart Trx f, down-regulates the STN7 activity, while earlier reports, based on *in vitro* experiments, showed the involvement of both f and m in the STN7 inhibition (Rintamäki *et al.*, 2000; Wunder *et al.*, 2013*b*). This discrepancy may be explained by the lack of specificity for different target enzymes that Trxs f and m usually revealed when *in vitro* studies were used (Geigenberger *et al.*, 2017).

### How Trx m overexpression induces STN7 inactivation

The current knowledge of Trx-mediated STN7 inactivation under HL supports two working hypotheses: (i) reduced Trx directly breaks the stromal disulfide bridge of STN7 thus interfering with ATP binding and leading to its inactivation (Rintamäki *et al.*, 2000; Puthiyaveetil, 2011); and (ii) reduction of the lumenal bridge involves a transthylakoid redox pathway for the delivery of reducing equivalents from the stroma to the lumen, with the CcdA and HCF164 proteins (Lennartz *et al.*, 2001; Page *et al.*, 2004) being the most suitable candidates (Dietzel *et al.*, 2008; Lemeille and Rochaix, 2010). Recent studies, however, speculate that the ROS generated in HL conditions might affect kinase folding in the thylakoid membrane, blocking its activity (Shapiguzov *et al.*, 2016). However, comparable ROS contents were shown in all genotypes (Supplementary Fig. S7), so ROS accumulation cannot account for the deactivation of STN7 in o/exTrxm plants. Within this context, our results would provide support *in vivo* that Trx m serves as the stromal source of reducing power for thiol-dependent regulation of STN7 activity.

It is well known that Stt7/STN7 interacts with the Cyt*b*_6_*f* complex by means of PetC (Lemeille *et al.*, 2009), an interaction that critically affects the kinase activity (Vener *et al.*, 1997; Zito *et al.*, 1999). Reverse genetic approaches have also unveiled a relevant role for PetC in state transitions and LHCII phosphorylation (Wollman and Lemaire, 1988; Wunder *et al.*, 2013*a*). However, our study showed that the amount of PetC protein in o/exTrxm plants was similar to (or even higher than) the quantity found in Wt plants (Supplementary Fig. S8), indicating that the STN7 deactivation in this line should not be attributed to PetC down-regulation. According to Shapiguzov *et al.* (2016), the movement of the lumenal PetC domain [which typically occurs during electron transfer within the Cyt*b*_6_*f* complex (Breyton, 2000)] could be coupled to a transitory dimer formation of the kinase, leading to its activation. The direct interaction between STN7 and PetC raises the possibility that the same redox system operates during regulation of their redox states. In this sense, it is known that the CcdA/HCF164 transthylakoid redox pathway is required for Cyt*b*_6_*f* assembly, with Trx m probably being the stromal electron donor for this system (Lennartz *et al.*, 2001; Page *et al.*, 2004; Motohashi and Hisabori, 2006, 2010). Therefore, Trx m overexpression could be causing the over-reduction of the STN7 lumenal Cys residues (by means of the CcdA/HCF164 pathway) with the consequent impact on transitory dimer formation, as has been shown after prolonged anaerobic treatment (Shapiguzov *et al.*, 2016). However, the fact that the overexpressed Trx m specifically associates with STN7 inside the chloroplast (Fig. 6), along with previous results showing that the mutation of the lumenal STN7 cysteines does not affect its interaction with Trx (Wunder *et al.*, 2013*b*), rather support the idea that Trx m causes changes in the redox state of Cys residues placed in the kinase domain, hampering its activity (Puthiyaveetil, 2011). Consistent with this, three m-type Trxs (m1, m2, and m4) have been identified in the peripheral fraction of the thylakoid membrane proteome from Arabidopsis chloroplasts, indicating that these isoforms are associated with the stromal side of the thylakoid membrane (Friso *et al.*, 2004). Some of the discrepancies with this model reported in Shapiguzov *et al.* (2016) could be explained by a dynamically buried/exposed Trx target site in STN7 as originally proposed by Rintamäki *et al.* (2000). With regard to the pulled-down PetC found in o/exTrxm plants (Fig. 6), it would be more likely to result from isolation of cross-linked endogenous protein complexes in chloroplasts, most probably by means of the CcdA/HCF164 redox pathway, than from a direct interaction with Trx m.

### Overexpression of Trx m shows altered protein complex composition and thylakoid architecture, as well as impaired photosynthetic performance in tobacco plants

The overexpression of Trx m in tobacco chloroplasts affected both the protein composition of photosynthetic complexes and the thylakoid structure, and consequently impacted photosynthesis (Figs 3–4; Table 1; Supplementary Figs S1–S2). The PSI–LHCII supercomplex (characteristic of state transitions) is formed when a subpopulation of pLHCII migrates from PSII to PSI so as to increase their optical absorption cross-section (Kouřil *et al.*, 2005; Pesaresi *et al.*, 2009; Järvi *et al.*, 2011; Allen, 2017). In this study, BN-PAGE fractionation of digitonin-solubilized thylakoid proteins revealed a clear correlation between LHCII phosphorylation and the amount of PSI–LHCII supercomplex and the related larger megacomplexes (Figs 1, 3). Thus, state transition-specific complexes were absent in o/exTrxm plants grown under LL conditions, reaffirming that in the absence of phosphorylation no migration of LHCII from PSII to PSI occurs, whereas they were abundant in o/exTrxf plants kept in the dark, for which a strong kinase activity is presumed (discussed above). Curiously, a small amount of PSI–LHCII supercomplex was perceived in both Wt and o/exTrxm plants in the dark (Fig. 3), in agreement with the residual LHCII phosphorylation level (Fig. 1C). Low STN7 activity in the dark has previously been reported in Arabidopsis, and was attributed to a stromal electron source triggering PQ pool reduction (Wunder *et al.*, 2013*a*). Therefore, our results indicate that the overexpressed Trx m must be reduced (using photosynthetic electrons provided by Fd during the day) to abolish LHCII phosphorylation efficiently, and this reinforces the importance of Trx m reductase activity in this process.

The overexpression of Trx m in tobacco chloroplasts likewise resulted in partial unstacking of grana (Fig. 4). Unstacking of thylakoid membranes may be induced by LHCII phosphorylation in the transition from state 1 to 2 (Chuartzman *et al.*, 2008) or by PSII core phosphorylation under HL stress (Tikkanen *et al.*, 2008; Herbstová *et al.*, 2012). However, in o/exTrxm plants, partial unstacking occurs in the absence of LHCII phosphorylation (plants blocked in state 1) and with a phosphorylation pattern of PSII core proteins similar to that of the Wt (Fig. 1A). Therefore, other factors such as the amount of LHCII or curvature thylakoid 1 (CURT1) proteins (Pribil *et al.*, 2014) may account for the observed phenotype. Along these lines, our results show a down-regulation of Lhcb1-2 in o/exTrxm plants (Supplementary Fig. S2), which could explain the observed rearrangement in the thylakoids. Accordingly, a similar thylakoid architecture phenotype has been reported from an Arabidopsis line deficient in Lhcb1 (Pietrzykowska *et al.*, 2014). Likewise, the light distribution difference in favor of PSI seen in o/exTrxm plants could easily be explained by the smaller PSII antenna size (Lhcb1-2 reduction), which agrees with the higher Chl *a*/*b* ratio of these plants (Table 2).

The observed decrease in LHCII proteins in o/exTrxm plants suggests a marked LHCII degradation process in this genotype. According to previous studies (Lindahl *et al.*, 1995; Yang *et al.*, 1998), dephosphorylated LHCII seems to be the preferred substrate for protease enzymes. Thus, the lack of LHCII phosphorylation in o/exTrxm plants suggests that protease enzymes may have degraded these proteins. There are several lines of evidence that FtsH, a metalloprotease essential for the repair of photodamaged D1, is also responsible for degradation of several LHCII apoproteins (Zelisko *et al.*, 2005; Luciński and Jackowski, 2013). Interestingly, redox control of the FtsH proteolytic activity has recently been demonstrated in *Chlamydomonas* (Wang *et al.*, 2017), suggesting that the overexpressed Trx m in tobacco chloroplasts might induce a reactivation of FtsH protease. Similarly, the PQ pool over-reduction in o/exTrxm plants could also down-regulate the transcription of *cab* genes (Escoubas *et al.*, 1995; Yang *et al.*, 2001), which may also contribute to the decreased LHCII level.

Energy required to sustain functioning of the photosynthetic apparatus requires an adjusted and co-ordinated light energy capture, which then needs to be transported through the photosynthetic electron chain and used in carboxylation. The absence of significant differences in stomatal opening or increases in substomatal CO_2_ concentration revealed that the impaired photosynthetic rates in o/exTrxm plants could not have been due to the availability of CO_2_ at the Rubisco carboxylation site. Instead, the reduced photosynthesis was due to a lower energetic status that negatively affected the photosynthetic machinery and CO_2_ fixation. In fact, our data show that the depleted ETR of o/exTrxm plants was the main factor explaining the lower *V*_Cmax_ and *J*_max_, which are both energy-demanding processes. In o/exTrxm plants, not only is the linear photosynthetic ETR impaired, as described in this work, but the cyclic ETR has also been reported as being markedly affected (Courteille *et al.*, 2013). In green algae, the absence of state transitions could also affect the photosynthetic cyclic electron flow (CEF; Finazzi *et al.*, 2002). However, it seems that the failure to undergo state transitions does not affect the CEF in the *stn7* mutant (Pesaresi *et al.*, 2009), implying that state transitions and CEF act independently in land plants. Rather, the absence of CEF in o/exTrxm plants could be related to the reduced thylakoid stacking observed in this line, as has recently been proposed (Johnson, 2018; Wood *et al.*, 2018). Overall, our results highlight the fact that the inhibited photosynthetic performance of o/exTrxm plants could be a consequence of depleted phosphorylated LHCII protein, which negatively affects the energetic status, photosynthetic machinery, and CO_2_ fixation in those plants. However, previous studies conducted with SNT7 mutants demonstrated that state transitions do not become critical for plant performance (Bellafiore *et al.*, 2005; Frenkel *et al.*, 2007), suggesting that factors other than STN7 deactivation should account for the observed o/exTrxm phenotype. In this sense, the overexpressed Trx m could be affecting the redox regulation of relevant chloroplast metabolic pathways such as C and N metabolism, thereby influencing the photosynthetic performance of this genotype.

### Conclusions

The present work provides the first *in vivo* evidence for the Trx-mediated STN7 inactivation in plants and contributes to elucidate the m-type Trx that specifically inhibits this kinase. Moreover, our results suggest that the overexpressed Trx m in tobacco chloroplasts might induce a reactivation of FtsH, with the concomitant degradation of LHCII (mainly the dephosphorylated forms), which in turn leads to alterations in thylakoid protein stoichiometry and ultrastructure. Both STN7 deactivation and the altered thylakoid architecture could account for the impaired photosynthetic performance of this genotype. In summary, the chloroplast behavior of o/exTrxm plants resembles that of plants exposed to HL stress, where LHCII phosphorylation is switched off and thylakoid architectural adaptation occurs so as to facilitate the repair of photodamaged PSII (Herbstová *et al.*, 2012). Altogether, our results may suggest a putative role for the Fd–Trx system, via Trx m, in governing the chloroplast response to HL intensities.

## Supplementary data

Supplementary data are available at *JXB* online.

Fig. S1. Lhcb2 protein content in thylakoid complexes.

Fig. S2. Analysis of Lhcb1-2 abundance.

Fig. S3. Generation of o/exTrxm-mut transplastomic tobacco plants.

Fig. S4. Trx m and f were efficiently precipitated in the pull-down assay.

Fig. S5. Pyridine nucleotide NADPH/NADP ratio in leaves.

Fig. S6. Abundance of ferredoxin.

Fig. S7. ROS accumulation in leaves.

Fig. S8. Abundance of PetC protein.

Supplementary Figure S1-S8
