# Supplementary material for: Overexpression of thioredoxin m in tobacco chloroplasts inhibits the protein kinase STN7 and alters photosynthetic performance
Source: J Exp Bot. 2018 Nov 21;70(3):1005–16. doi: 10.1093/jxb/ery415 (PMC6363096; doi:10.1093/jxb/ery415)
Supplement: Supplementary Figure S1-S8 [file ery415_suppl_supplementary-figure-s1-s8.pdf]

Thioredoxin m overexpression in tobacco chloroplasts inhibits the protein kinase STN7 and alters photosynthetic performance

M. Ancín[1], A. Fernández-San Millán[1], L. Larraya[1], F. Morales[1 2], J. Veramendi[1], I. Aranjuelo[1] and I. Farran[1]

[1]Instituto de Agrobiotecnología (IdAB), Universidad Pública de Navarra-CSIC-Gobierno de Navarra, Campus Arrosadia, 31006, Pamplona, Spain; [2]Estación Experimental de Aula Dei (EEAD). CSIC. Dpto. Nutrición Vegetal. Apdo. 13034. 50080. Zaragoza, Spain.

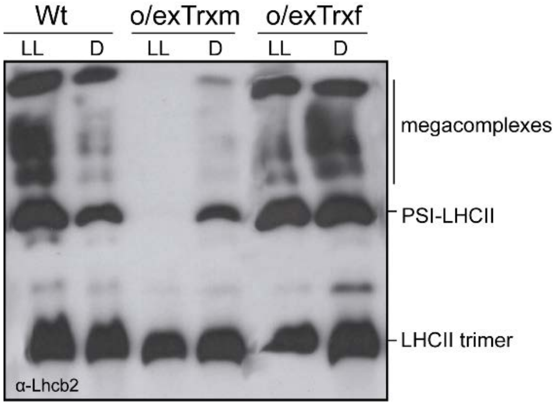

Fig. S1. Lhcb2 protein content in thylakoid complexes. After thylakoid complexes separation by BN-PAGE, proteins were transferred to PVDF membrane and immunoblotted against Lhcb2 antibody (Agrisera AB, Vännäs, Sweden). LL, low light; D, dark.

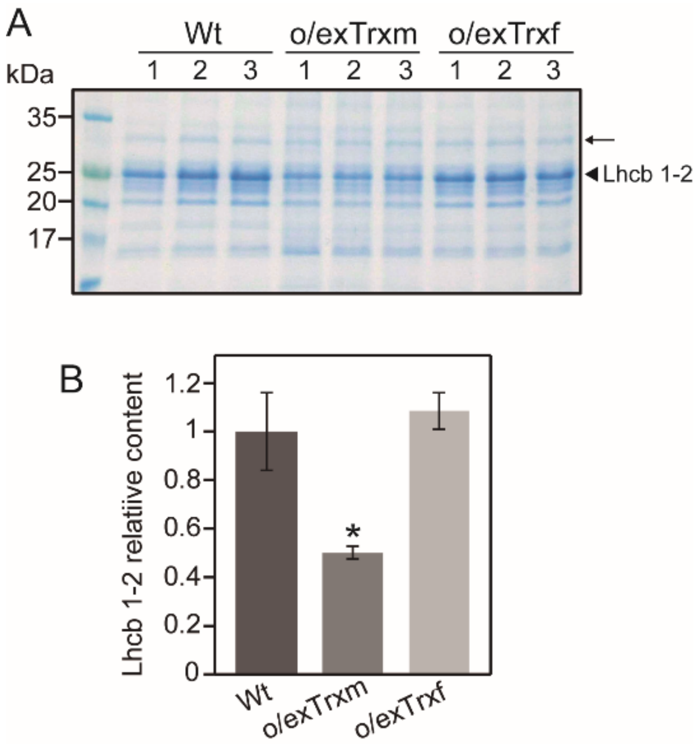

Fig. S2. Analysis of Lhcb1-2 abundance. A) Thylakoid proteins (30 µg) were separated by SDS-PAGE (13%) and visualized by Coomassie Blue R-250 staining. Identification of protein Lhcb1-2 was performed in accordance with Rumak *et al*. (2012, *BMC Plant Biology,* 12:72). B) Semiquantitative analysis of the Lhcb1-2 protein amount. Coomassie gel in A was analyzed with GeneTools software. Protein levels in Wt, o/exTrxm and o/exTrxf plants are shown relative to the levels of a constitutively expressed protein (indicated with an arrow). Data are given as means ± SE of three biological replicates. Statistical significance relative to Wt plants are indicated by asterisk ($P \leq 0.05$, Student's t test).

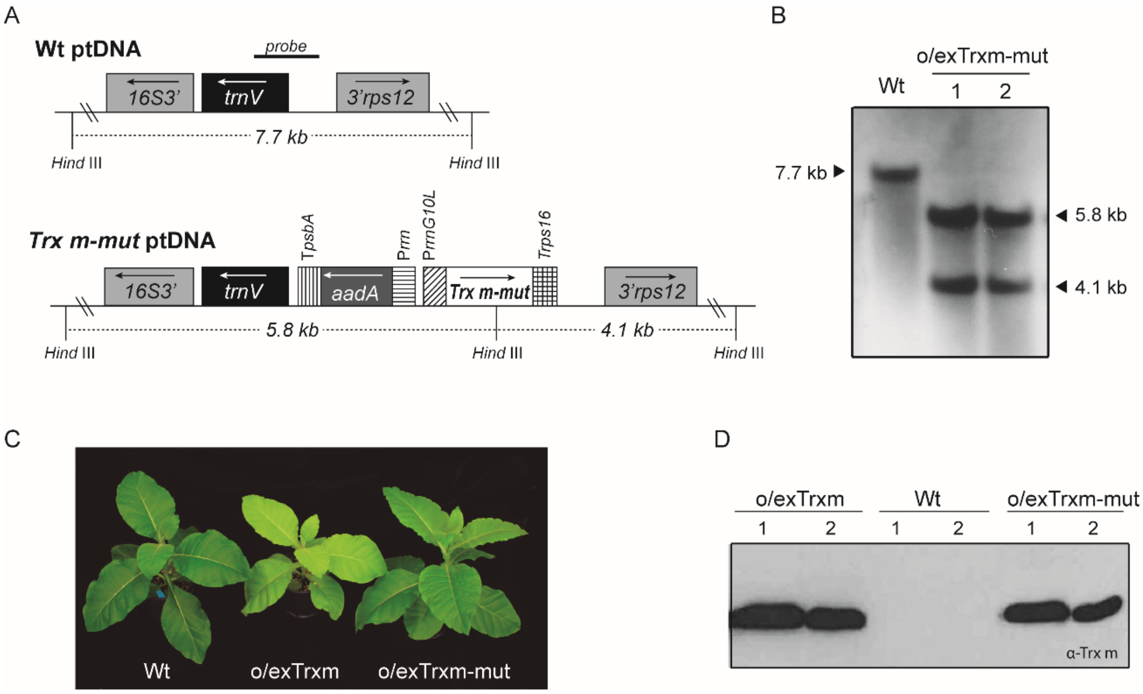

Fig. S3. Generation of o/exTrxm-mut transplastomic tobacco plants and analysis of the transgene expression. Transplastomic plants were generated by chloroplast transformation as previously described (Sanz-Barrio *et al*., 2013, *PBJ,* 11:618). A) Map of the Wt and transformed Trxm-mut plastid genome. The *Trxm-mut* gene (generated by site-directed mutagenesis using the following oligonucleotides: forward-5′-CTCCGTGGAGTGGTCCAAGCCGAATG-3′ and reverse-5′-CATTCGGCTTGGACCACTCCACGGAG-3′, which included a double change to replace Cys37 and Cys40 with serine), driven by the *rrnG10L* promoter and *rps16* terminator, was cloned into the intergenic region between *16S3′/trnV* and *3'rps12* genes in the duplicated inverted repeat region. The arrows in the boxes show the direction of transcription. The selectable marker gene *aadA* is driven by the *rrn* promoter. The probe for the Southern blot is shown over the corresponding sequence. The sizes of the predicted bands after DNA digestion with *HindIII* are indicated. *16S3', trnV and 3'rps12*: original sequences of the chloroplast genome; *aadA*: aminoglycoside 3'-adenylytransferase gene; P*rrn*: *16S*rRNA promoter and 5'-untranslated region; P*rrnG10L*: *16S* rRNA promoter fused to the leader region of the bacteriophage T7 gene *10*; T*psbA*: terminator region of the *psbA* gene; T*rps16*: terminator region from the chloroplast *rps16 gene.* B) Integration of the *Trxm-mut* gene into the chloroplast genome and homoplasmy confirmation by Southern blot analysis. Total DNA (10 µg) of two Trxm-mut plants and a Wt control were digested with HindIII and probed. C) Visual appearance of Wt, o/exTrxm and o/exTrxm-mut plants. Representative photographs are shown. D) Trx m mutated variant expression levels in o/exTrxm-mut plants are similar to those of Trx m in o/exTrxm transplastomic plants. Immunoblot analyses of total protein extracts (10 µg) from mature leaves were performed. Specific anti-Trx m antibody was used (Sanz-Barrio *et al*., 2011, *Plant Biotechnol J*, 9:639).

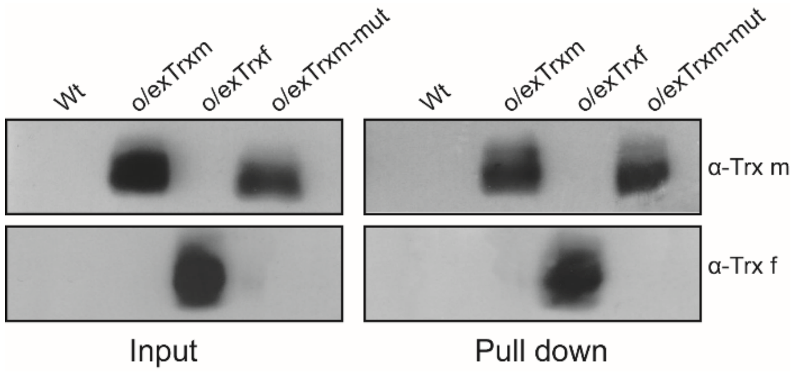

Fig. S4. Trx m and f were efficiently precipitated in the pull down assay. We analyzed the overexpressed Trx m or f amount in the pulled-down fractions after incubation of Ni-NTA beads with chloroplast lysates from Wt, o/exTrxf, o/exTrxm and o/exTrxm-mut plants. After washing the beads, bound proteins were eluted by boiling. Input fractions and pulled-down extracts were analyzed by western blot using Trx m and f-specific antibodies (Sanz-Barrio *et al*., 2011, *Plant Biotechnol J*, 9:639). Trx m was only detected in the eluted fraction from o/exTrxm and o/exTrxm-mut lines, whereas Trx f protein was present in the eluted fraction from o/exTrxf transgenic extracts. The wild-type endogenous Trx f/m proteins were not detected in these blots because of the large differences in the expression level (Sanz-Barrio *et al*., 2013, *Plant Biotechnol J*, 11:618).

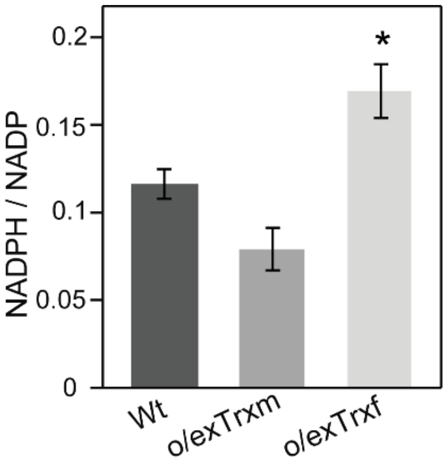

Fig. S5. Pyridine nucleotide NADPH/NADP ratio in leaves. Leaves from Wt, o/exTrxm and o/exTrxf plants were harvested after 4 h illumination and pyridine nucleotides measured. Values are mean ± SE of measurements of 5 biological replicates. Statistical significance compared to Wt plants is indicated by asterisks ($P<0.05$, Student's *t*-test).

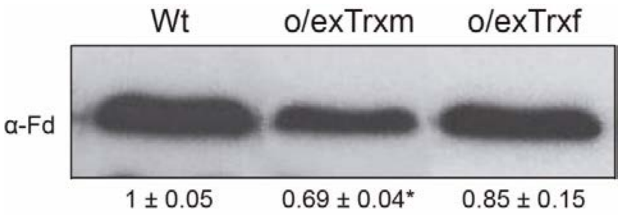

Fig. S6. Abundance of ferredoxin (Fd), the main electron acceptor from PSI. The Fd content in Wt, o/exTrxm and o/exTrxf plants was determined by western blot. Total proteins (50 µg) were separated by SDS-PAGE (18%). Immunoblotting was performed using specific Fd antibody (Agrisera AB) at a dilution of 1:1000. Numbers below the blot show signal strength in the Trx lines relative to Wt ± standard error obtained from analysis of protein extracts of three plants in three biological replicates. Statistical significance compared to Wt plants is indicated by asterisks ($P<0.05$, Student's *t*-test).

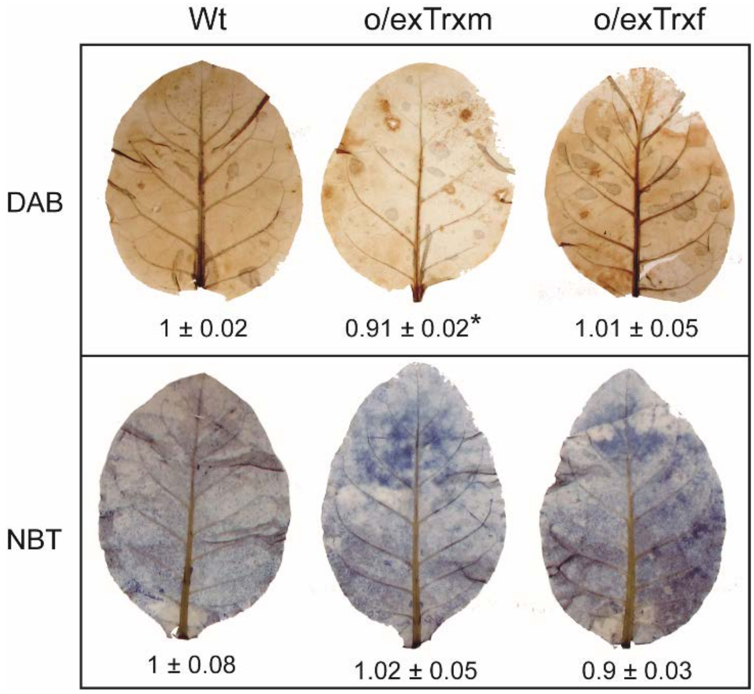

Fig. S7. ROS accumulation in leaves of tobacco plants. Leaves from 4-week-old Wt, o/exTrxm and o/exTrxf plants grown under 16 h photoperiod were harvested after 7 h of illumination. The upper panel shows 3,3′-diaminobenzidine (DAB) *in situ* histochemical staining for hydrogen peroxide (H_2O_2) production. The lower panel illustrates nitroblue tetrazolium chloride (NBT) *in situ* histochemical staining for superoxide (O_2^-) detection. Staining was carried out according to Lu *et al*. (2011, *Plant Cell*, 23:1861) with some modifications. DAB staining: detached leaves were vacuum-infiltrated with 1mg/ml DAB water solution, pH 3.8, for 2 min and incubated in the same solution under growth light for 90 min. NBT staining: detached leaves were vacuum-infiltrated with 10mM potassium phosphate buffer pH 7.8 containing 10mM NaN_3 for 2 min and incubated in 0.1% NBT for 90 min under growth light. In both cases, pigments were removed by boiling 5 min in acetic acid-glycerol-ethanol (1/1/3) and leaves were immediately photographed. The quantification of the histochemical staining intensity was performed with Adobe Photoshop CS software. The experiment was conducted three times with similar results. For each treatment, a representative infiltrated leaf is shown. Numbers under pictures indicate quantitative measurements as mean ± SE of 6 biological replicates. Statistical significance compared to Wt plants is indicated by asterisks ($P<0.05$, Student's *t*-test).

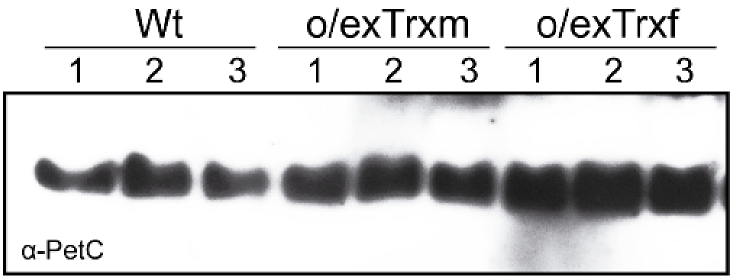

Fig. S8. Abundance of PetC protein. Western blot analysis was performed to determine the amount of PetC protein in Wt, o/exTrxm and o/exTrxf plants. Similar amounts of protein were loaded for each line, separated on 15% SDS-PAGE containing 6M urea, transferred to a PVDF membrane and immunoblotted against a PetC antibody (Agrisera AB) at a dilution of 1:5000.
